# Case Report: A case series of using ultrasound-guided continuous parasacral ischial plane blocks to promote ulcer healing and pain relief in people with diabetic foot ulcers

**DOI:** 10.3389/fendo.2025.1580568

**Published:** 2025-08-25

**Authors:** Peng Ye, Xuan Pan, Jing Zhuang, Hanliang Fan, Xiaochun Zheng, Ting Zheng

**Affiliations:** ^1^ Department of Anesthesiology, Shengli Clinical Medical College of Fujian Medical University, Fujian Provincial Hospital, Fuzhou University Affiliated Provincial Hospital, Fuzhou, China; ^2^ Fujian Provincial Key Laboratory of Emergency Medicine, Fujian Provincial Key Laboratory of Critical Care Medicine, Fujian Provincial Co-Constructed Laboratory of “Belt and Road,” Fujian Emergency Medical Center, Fuzhou, China; ^3^ Department of Plastic and Burn, Shengli Clinical Medical College of Fujian Medical University, Fujian Provincial Hospital, Fuzhou University Affiliated Provincial Hospital, Fuzhou, China

**Keywords:** diabetic foot ulcers, pain relief, parasacral ischial plane block, ultrasound-guided, angiogenesis

## Abstract

**Background:**

Diabetic foot ulcers (DFU) are a prevalent complication of diabetes, leading to significant morbidity, mortality, and amputation rates. Chronic non-healing DFU often result from peripheral neuropathy, microvascular issues, and infection, with poor blood and oxygen supply being critical factors in delayed healing. The development of new treatments to promote blood supply and accelerate ulcer healing is a significant area of research for DFU management.

**Methods:**

In this case series, nine patients with chronic diabetic ulcers received ultrasound-guided continuous parasacral ischial plane block (CPIPB) for 2 weeks, combined with standard care (SOC) for 12 weeks. Our objective was to assess the reduction in wound size and the rate of complete healing 12 weeks after the start of treatment.

**Results:**

Among the nine patients, six achieved complete healing of their diabetic foot ulcers, while the remaining three showed significant improvement with more than a 50% reduction in the initial ulcer area. This approach not only provided analgesia but also increased foot skin temperature and blood supply in the lower limb arteries, potentially promoting angiogenesis and revascularization. Local anesthetic leakage at the catheter insertion site occurred in two cases during CPIPB.

**Conclusion:**

CPIPB can promotes diabetic foot ulcer healing while providing effective analgesia in the lower limbs. This approach may offer a valuable complement to conventional treatments, particularly for patients with severe pain.

## 1 Introduction

Diabetic foot ulcers (DFU), a major concern worldwide, are prevalent complications of diabetes, mainly affecting long-term diabetic patients (1). Chronic non-healing DFU often cause non-traumatic amputations due to peripheral neuropathy, microvascular issues, and diabetic ulcer infections. Poor blood and oxygen supplies are critical factors for delayed healing (2). The chronic hyperglycemic state of patients with diabetes causes damage to blood vessels, which affects blood supply, especially to the lower extremities (3). DFU can result in severe morbidity, mortality, and amputation if not properly treated.

Current treatments for DFU include glycemic and diet control, conventional therapy for ulcers, resection of chronic wound/ulcer, and revascularization (4). However, these approaches are not always successful in promoting ulcer healing, especially if circulation is severely impaired. Therefore, the development of new treatments to promote blood supply and accelerate ulcer healing has garnered attention in DFU research. Nerve block has a sympathectomy-like vasodilation effect, which can dilate blood vessels and relieve pain (5).Although single-injection blocks targeting the sciatic or femoral nerve are widely utilized in clinical settings for analgesia, their anatomical remoteness from the sympathetic chain results in transient and non-sustained vasodilation. This inherent limitation likely compromises their therapeutic potential in improving diabetic foot ulcer healing (6). Continuous parasacral ischial plane block (CPIPB), an innovative technique of continuous sacral plexus block, can increase foot skin temperature and blood flow speed in the lower limb arteries (7), which may have therapeutic potential for DFU. In this study, we aimed to investigate the effects of CPIPB on DFU healing.

## 2 Research design and methods

Between June and November 2024, we conducted a case series study involving nine patients with diabetic foot ulcers (ulcer area > 1 cm², UTC grade 1-3). These patients had not shown signs of healing for at least three months despite conventional treatments. Informed consent was obtained from all participants. Patients with unstable hemodynamics due to ischemic heart disease or coagulation disorders, as well as those on anticoagulant and/or non-steroidal anti-inflammatory drug (NSAID) therapy, were excluded. Additionally, patients with the following characteristics were also excluded: platelet count below 150,000/mm³, uncontrolled diabetes mellitus, allergic to ropivacaine and patients who are pregnant or planning to become pregnant, in order to optimize the outcome of the treatment.

**Figure 1:**
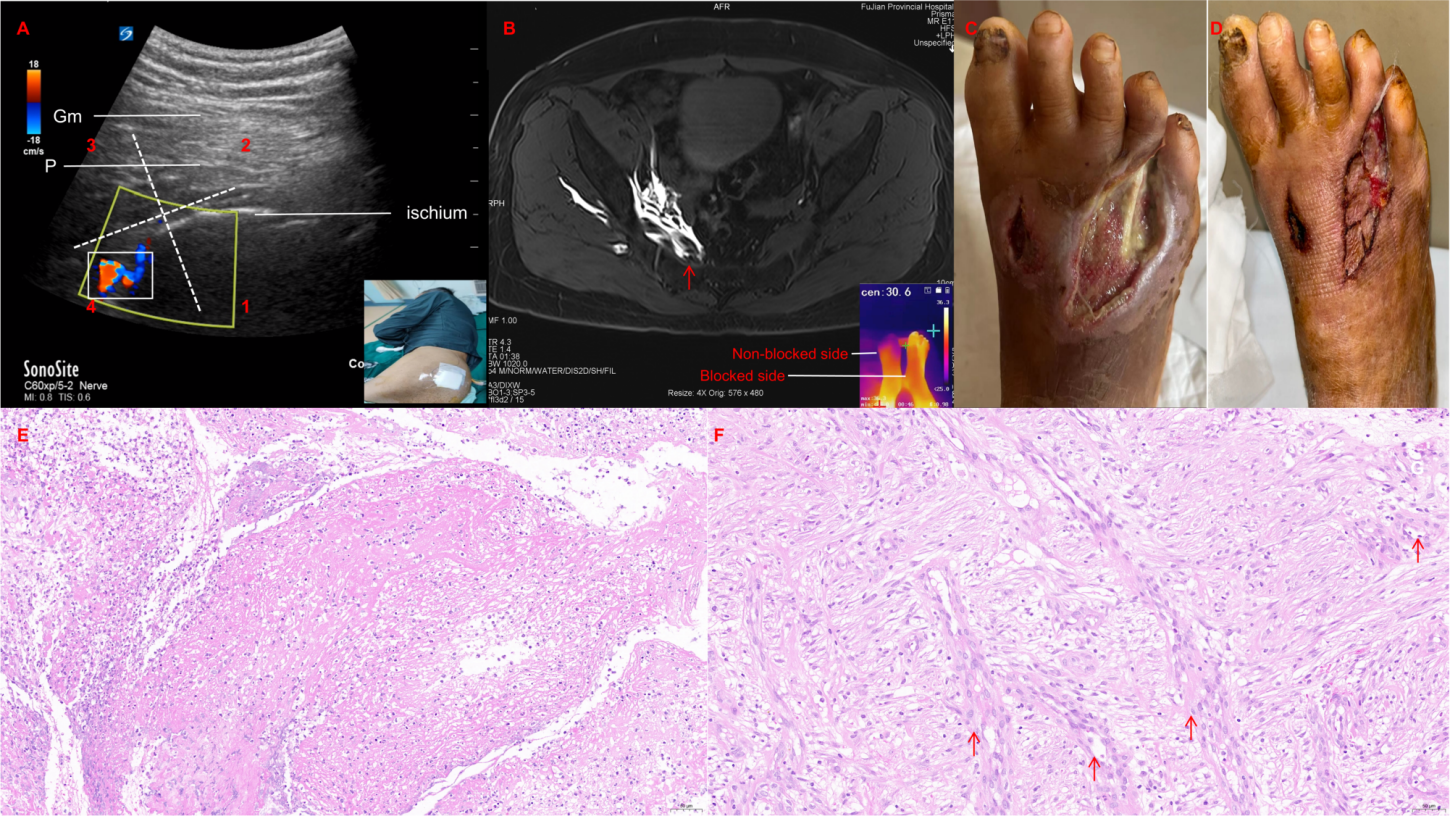
Continuous parasacral ischial plane block (CPIPB) and ulcer healing. **(A)** Ultrasound-guided CPIPB. The white box represents ultrasound Doppler monitoring while the catheter is placed. **(B)** The patient’s foot skin temperature increased after the block, and magnetic resonance imaging showed that the sacral sympathetic nerve was blocked by the local anesthetic at the position indicated by the red arrow. **(C)** Pre-treatment images from one patient show limited blood supply to the wound tissue and slow granulation tissue growth. **(D)** Post-treatment images from the same patient demonstrate increased blood supply, extensive granulation tissue proliferation, re-epithelialization, and accelerated healing after 2-weeks CPIPB. **(E)** Hematoxylin-eosin (HE) staining before CPIPB treatment. Increased inflammatory cell infiltration and decreased neovascularization were observed. **(F)** HE staining after CPIPB treatment showed that inflammatory cell infiltration decreased, while fibro-proliferation and neovascularization increased. The position of the red arrow indicates the location of the neovascularization.

Each patient was managed in a dedicated multidisciplinary diabetic foot ulcer clinic at a university hospital and received continuous parasacral ischial plane block (CPIPB) two weeks, alongside standard of care (SOC) 12 weeks. The specific treatment process includes patients undergoing parasacral ischial plane catheterization by the same treatment team in accordance with the study method proposed by Ye et al. (7). After successful catheterization, 0.2% ropivacaine was administered at a rate of 5 ml/h via a microinjection pump for 14 days (**Figure 1A**). As part of the treatment plan, the SOC treatment included glycemic control and proper debridement, and the wound was covered with ordinary Vaseline gauze.

The primary outcome of the study was the rate of complete ulcer healing observed in patients at week 12. The ulcer area was evaluated upon entry into the study and at a week 12 interval after the first day CPIPB injection. Additionally, the rate of complete wound healing (defined as the wound complete epithelialization) was quantified (8). The ulcers were assessed using the PUSH scale (9) at the beginning of the treatment (T0) and at the week 12 follow-up(T12). Other secondary measures included the patients’ pain scores, ulcer area changes, bilateral foot skin temperature differences, peak systolic velocity (PSV) of the anterior tibial artery (ATA), and potential complications arising at week 12.

The database was established using Microsoft Excel 2019, and data analysis was performed using SPSS version 25.0. For quantitative data, the Shapiro-Wilk test was used to assess normality, and Levene’s test was conducted to evaluate homogeneity of variance. Data(Normality of Differences) that met the assumptions of normality were expressed as mean (standard deviation, SD), and comparisons were made using the paired-samples t-test. Data that did not meet these assumptions were presented as median (interquartile range), and comparisons were performed using the Wilcoxon Signed-Rank Test. Categorical variables were presented as frequencies (percentages). The ulcer areas at various time points were subjected to analysis utilizing the Friedman test. Subsequently, pairwise comparisons were conducted, and the p-values were adjusted using the Bonferroni correction method. *P*<0.05 was considered to indicate statistical significance. This case series has been reported in line with the PROCESS Guideline (10).

## 3 Results

The study enrolled 9 treated patients (3 males, 6 females) aged 36–84 years (median 69). Four patients had ulcers on the dorsum of the foot, while five patients had ulcers on the plantar surface. The ulcer area demonstrated a mean baseline size of 4.6 cm^2^ (Standard deviation 1.2), with no gangrenous ulcers observed. The UTC classifications of the nine patients with diabetic foot ulcers were as follows: 1B (1 case), 2A (1 case), 2B (2 cases), 2C (2 cases), 3A (1 case), and 3B (2 cases). Among them, a total of five patients (1B, 2B, 3B) had infections in their ulcers, while two patients (2C) had severe ischemia associated with their ulcers. the baseline patient characteristics are shown in **Table 1** (11). All nine patients received a 2-week course of CPIPB combined with 12-weeks SOC therapy. At the 12-week follow-up, six patients (66.7%) achieved complete ulcer healing, while one of the six patients experienced recurrence after complete healing. Diabetic foot ulcer area was significantly reduced at week 12 compared to baseline (T0) and week 2 (T2) and there was a statistically significant difference in the ulcer area between T12 and T0 (using the Friedman test).

**Table 1:** Demographic characteristics demographic.

Parameters	Patients treated (n=9)
Age, years	69 (67, 72)
Sex	
Male, %	3 (33.3%)
Female, %	6 (66.7%)
BMI, kg/m2	25.8 (1.6)
Type of DM	
Type 1	3 (33.3%)
Type 2	6 (66.7%)
HbA1c, mmol/l	69 (8.4)
Duration of diabetes, years	16.7 (5.5)
Ulcer location	
Dorsum foot skin	4 (44.4%)
Plantar foot skin	5 (55.6%)
UTC grade	
1B	1 (11.1%)
2A	1 (11.1%)
2B	2 (22.2%)
2C	2 (22.2%)
3A	1 (11.1%)
3B	2 (22.2%)
Albumin level, g/L	42.7 (5.7)
Diabetes complicated	
Peripheral neuropathy	2
Diabetic nephropathy	2
Diabetic retinopathy	1
Hypertension	5
Pulse status of dorsal pedal arteries	
Normal	4 (44.4%)
Diminished	4 (44.4%)
Absent	1 (11.1%)
Inflammatory biomarkers	
Procalcitonin (PCT) (Normal:<0.05mg/L)	4.5 (1.8)ng/mL
C-Reactive Protein (CRP) (Normal:<5mg/L)	123.8 (63.8)mg/L

The value Summary was present as mean ± SD or median (IQR)or n (%)

IQR, interquartile range.

The PUSH (Pressure Ulcer Scale for Healing) scores significantly decreased from 10 (interquartile range: 7.5 to 10) before treatment to 0 (interquartile range: 0 to 5.5) after 12 weeks of treatment (*P* = 0.007).The secondary observational indicators showed that the mean VAS score for pain significantly decreased from 6.9 (SD 1.6) before treatment to 1.8 (SD 1.1) at the 12-week follow-up (*P* < 0.001). Additionally, notable alterations in the skin temperature of the dorsum of the foot in the blocked lower limb, pronounced elevation of skin temperature at the blocked ulcer site, and a marked increase in the peak systolic velocity (PSV) of the anterior tibial artery (ATA) were observed (**Table 2**). One patient provided informed consent and underwent hip magnetic resonance imaging (MRI) to assess ropivacaine diffusion. During CPIPB, MRI was performed on a Siemens 3T Magnetom Prisma Fit scanner (Siemens). Using T1 VIBE Dixon sequence with a scanning thickness of 2 mm. MRI indicated that the administration of local anesthetics improved lower-limb perfusion by inhibiting the activity of the sacral sympathetic nerves (**Figure 1B**). The granulation tissue of the ulcer wound proliferated extensively and the wound was re-epithelialized, indicating signs of accelerated healing (**Figures 1C, D**). Histological analysis of the ulcer site pre- and post-blockade demonstrated substantial enhancement in both inflammatory reparative processes and angiogenesis (**Figures 1E, F**). There were no apparent complications during treatment, and only two patients had insertion site leakage, which was resolved by catheter positioning and resuturing.

**Table 2:** Treatment outcomes data.

Parameters	Patients treated (n=9)	p-value
Ulcer area (cm²)		
T0^a^	4.6 (3.4, 5.4)	a, b: 0.102 a, c: <0.001* b, c: 0.102
T2^b^	3.6 (2.6, 5.1)	
T12^c^	0 (0, 1.9)	
Ulcer healing rate		
T2	0 (0%)	–
T12	6 (66.7%)	–
Time to ulcer healing (weeks)	10.8 (4.9)	–
Scores of PUSH scale		
T0	10 (7.5, 10)	0.007*
T12	0 (0, 5.5)	
Ankle-brachial index (ABI)		
T0	0.89 (0.08)	0.114
T12	0.93 (0.10)	
Temperature difference between limbs (°C)		
T0	-0.2 (-0.35, 0.2)	0.007*
T12	0.7 (0.55,1.0)	
ATA PSV		
T0	33.5 (10.1)	<0.001*
T12	44.3 (9.5)	
VAS		
T0	6.9 (1.6)	<0.001*
T12	1.8 (1.1)	

The analysis of ulcer area using the Friedman test showed that the repeated measures *P* value was <0.001, indicating a significant difference in ulcer area between the time points before and after treatment (T0, T2, T12). Further pairwise comparison results were as follows: the *P* value for comparison between a and b was 0.102; the P value for comparison between a and c was <0.001; and the *P* value for comparison between b and c was 0.102. CPIPB, continuous parasacral ischial plane block; VAS, visual analog scale; PSV, peak systolic velocity; ATA, anterior tibial artery;T0, at the beginning of the treatment;T2, at the week 2 follow-up;T12, at the week 12 follow-up. ^*^indicates *P*< 0.05, which was performed by paired t-test or non-parametric test.

## 4 Discussion

This case series represents the first report of CPIPB application in managing diabetic foot ulcers, demonstrating facilitated wound healing and concurrent alleviation of lower extremity neuropathic pain. In the future, CPIPB could serve as a significant adjuvant therapeutic approach for enhancing ulcer healing in individuals with diabetic foot.

Current literature suggests that inadequate blood flow significantly hampers the healing processes of diabetic foot ulcers. Insufficient perfusion can lead to ischemia, which compromises the delivery of essential nutrients and oxygen to the affected tissues, thereby impairing the cellular functions necessary for wound healing (12). Moreover, the inflammatory response, which is crucial for healing, can be disrupted, further complicating the recovery process (13).Prior studies have emphasized the importance of assessing peripheral artery disease (PAD) in patients with DFUs, as the presence of PAD correlates with higher rates of non-healing ulcers and subsequent amputations (14).

The message conveyed through this case report is that addressing lower limb blood supply is paramount in the management of diabetic foot ulcers. Inadequate healing observed in patients with DFU is strongly associated with compromised blood flow in the lower extremities, particularly in the anterior and posterior tibial arteries, which are susceptible to vascular stenosis. Progression of this condition may extend to the arterial system proximal to the knee joint. Nerve innervation of these vessels originates from the sacral sympathetic nerve, specifically the grey ramus communicans of the S2-S4 nerve trunk (15). Our MRI findings support the hypothesis that CPIPB distribution of the local anesthetic around the sacral sympathetic nerve results in sustained vasodilation in the lower limbs and enhanced healing processes, which may be related to the promotion of angiogenesis and revascularization. Angiogenesis is an important physiological response that is associated with diabetic skin wounds (16).The study found a significant increase in angiogenesis in treated ulcer wounds. Although the specific mechanism is currently unclear, it may be related to the improved blood supply and oxygenation.

Current treatment strategies for DFU involve only symptom management, with limited attention given to the cause of pain (17). Patients with DFU frequently experience intense pain in the advanced stages and often find conventional analgesics ineffective. The sacral plexus nerve, encompassing branches such as the sciatic nerve and superior and inferior gluteal nerves, plays a crucial role in innervating common pain sites associated with DFU, particularly below the ankle joint. Therefore, during the CPIPB, it can quickly relieve the patient’s lower limb pain. Notably, sustained pain relief persisted through 12-week posttreatment follow-up despite the 2-week intervention course of continuous sacral plexus block. This delayed therapeutic effect may be attributed to blockade-induced ischemic vasodilation, which potentially enhances neural perfusion thereby alleviating neuropathic pain associated with diabetic neuropathy (18).Meanwhile, low-concentration ropivacaine has the advantage of separating sensory-motor blocks, as it minimally affects motor function while maintaining a high safety profile (19).

There are two surgical approaches for individuals with DFU and lower-limb vascular stenosis in clinic: endovascular intervention and lumbar sympathectomy radiofrequency ablation surgery (20). While endovascular interventions, such as arterial angioplasty and stent implantation, demonstrate significant therapeutic efficacy in alleviating mechanical obstructions—particularly in patients with ischemic diabetic foot ulcers—their effectiveness may be constrained by several factors. These include the condition of the vasculature (such as the degree of blockage and calcification), the fragility of the patient, the concomitant degree of tissue loss and infection (limb severity), and the anatomic complexity (21, 22). Moreover, the capacity of these interventions to ameliorate microvascular dysfunction appears to be limited (23, 24). Importantly, addressing the pain associated with diabetic foot ulcers often necessitates a comprehensive treatment approach that involves multidisciplinary team collaboration (25). Consequently, in addition to revascularization, it is essential to employ supplementary therapeutic strategies aimed at improving microcirculation disorders and alleviating pain. Conversely, lumbar sympathectomy radiofrequency ablation can dilate lower-limb vessels, but its effects are gradual and irreversible. It may lead to irreversible complications such as ejaculatory dysfunction and abnormal lower-limb sensations. When patients with DFU experience severe pain and are hesitant to pursue surgical intervention, CPIPB may be considered a complementary approach for pain management and therapeutic intervention.

While the CPIPB technique was effective in pain relief and ulcer healing in our case series, concerns remain about potential catheter infections with long-term nerve block. However, no signs of infection were observed after careful nursing. The safety of long-term CPIPB for DFU patients remains a critical clinical concern. The safety of long-term CPIPB is also a concern. In this study, the optimized CPIPB protocol (low-concentration ropivacaine at a slowly infusion speed) achieved therapeutic vasodilation without inducing local anesthetic systemic toxicity (LAST) throughout the 14-day blockade period and 12-week follow-up. However, subsequent investigations incorporating serum drug concentration monitoring and long-term nerve conduction studies are warranted to fully characterize the safety parameters of this intervention.

## 5 Limitations

Our study possesses certain limitations that warrant consideration. Firstly, while our findings suggest that CPIPB may facilitate the healing of DFU, the scope of our investigation was limited to assessing the pulse status of dorsal pedal arteries and the ankle-brachial index. These parameters may imply that the lower extremity ulcers in our patients were non-ischemic. Consequently, further research is necessary to elucidate the efficacy of CPIPB in various DFU types, particularly in ischemic ulcers with compromised vascular conditions. Secondly, the absence of CT or MRI angiography in our study precluded a comprehensive assessment of the vascular status of the lower extremities. Future studies should address this gap to enhance the understanding of CPIPB’s role. Finally, it is crucial to acknowledge that our research is derived from a small-sample case report study. Therefore, large-sample, multicenter randomized controlled trials are necessary to further elucidate the long-term therapeutic efficacy and underlying mechanisms of CPIPB in the management of diabetic foot ulcers.

## 6 Conclusions

CPIPB can promotes diabetic foot ulcer healing while providing effective analgesia in the lower limbs. This approach may offer a valuable complement to conventional treatments, particularly for patients with severe pain.

## Data Availability

The original contributions presented in the study are included in the article/supplementary material. Further inquiries can be directed to the corresponding authors.
